# Monkeypox Patients Living with HIV: A Systematic Review and Meta-Analysis of Geographic and Temporal Variations

**DOI:** 10.3390/epidemiologia4030033

**Published:** 2023-09-04

**Authors:** Aravind P. Gandhi, Bijaya K. Padhi, Mokanpally Sandeep, Muhammad Aaqib Shamim, Tarun K. Suvvari, Prakasini Satapathy, Abdelmonem Siddiq, Ranjit Sah, Sarvesh Rustagi, Zahraa H. Al-Qaim, Jagdish Khubchandani

**Affiliations:** 1 Department of Community Medicine, ESIC Medical College & Hospital, Sanathnagar, Hyderabad 500038, India; aravindsocialdoc@gmail.com; 2 Department of Community Medicine and School of Public Health, Postgraduate Institute of Medical Education and Research, Chandigarh 160012, India; bkpadhi@gmail.com; 3 School of Medical Sciences, University of Hyderabad, Hyderabad 500046, India; sandeepmokanpally@gmail.com; 4 Department of Pharmacology, All India Institute of Medical Sciences, Jodhpur 342005, India; aaqibsh@gmail.com; 5 Medical School, Rangaraya Medical College, Kakinada 533001, India; drtarunsuvvariresearch@gmail.com; 6 Global Center for Evidence Synthesis, Chandigarh 160036, India; prakasini.satapathy@gmail.com; 7 Faculty of Pharmacy, Mansoura University, Mansoura 35516, Egypt; abdelmonemalsaid555@std.mans.edu.eg; 8 Department of Microbiology, Tribhuvan University Teaching Hospital, Institute of Medicine, Kathmandu 44600, Nepal; ranjitsah@iom.edu.np; 9 Department of Microbiology, Dr. D. Y. Patil Medical College, Hospital and Research Centre, Dr. D. Y. Patil Vidyapeeth, Pune 411018, India; 10 School of Applied and Life Sciences, Uttaranchal University, Dehradun 248007, India; sarveshrustagi@uumail.in; 11 Department of Anesthesia Techniques, Al-Mustaqbal University College, Hillah 51001, Iraq; zahraahaleem@uomus.edu.iq; 12 Department of Public Health Sciences, New Mexico State University, Las Cruces, NM 88003, USA

**Keywords:** zoonosis, monkeypox, HIV, infection, meta-analysis

## Abstract

This index meta-analysis estimated the pooled prevalence of human immunodeficiency virus (HIV) among individuals with monkeypox (mpox) globally. We searched seven databases: PubMed, Scopus, Web of Science, EMBASE, ProQuest, EBSCOHost, and Cochrane, for human studies published in English till 4 January 2023, as per International Prospective Register of Systematic Reviews (PROSPERO) registration protocol (CRD42022383275). A random effects regression model was used to estimate the pooled prevalence owing to high heterogeneity. The risk of bias in the included studies was assessed using the National Heart, Lung, and Blood Institute (NHLBI) quality assessment tool. The systematic search yielded 677 articles; finally, 32 studies were found eligible for systematic review and 29 studies for meta-analysis. The pooled prevalence of HIV infection was 41% (95% confidence interval [CI], 35–48). All studies were rated as fair or good quality. Studies from Europe and North America reported a high prevalence of HIV infection among individuals with mpox- 41% (95% CI 33–49) and 52% (95% CI 28–76), respectively, while studies from Nigeria, Africa reported a relatively low prevalence of HIV infection of 21% (95% CI 15–26)**.** A history of sexual orientation and sexual partners in the last 21 days must be taken from individuals with mpox to identify the potential source and contacts for quarantining and testing them.

## 1. Introduction

Most mpox (previously known as monkeypox) cases were reported from central and west Africa in the past [1]. However, in May 2022, the disease began to emerge across the world in multiple countries. It was declared a “public health emergency of international concern’ by the World Health Organization (WHO) in 2022 [2]. Among all known transmission routes, a potential sexual route has been reported among 82.1% (16518/20126) of individuals with mpox [3]. Within mpox virus (MPXV), there are two distinct clades, namely clade I (formerly known as Central Africa strain) and clade II (formerly known as West African strain). Clade II encompasses two phylogenetically distinct subclades, IIa and IIb. The sub-clades are however distinct, with a differential evolution from an ancestor which dates back centuries [4]. The 2022 multicountry outbreak of mpox has been attributed to clade IIb. Pre-2022 outbreak of mpox was mostly associated with close contact through the skin lesions and respiratory route of transmission [5]. In contrast, sexual intercourse has been identified as a major risk factor for the 2022 multicountry outbreak. Close contact happening during sexual intercourse or as sexually transmitted infection due to the act are the potential routes of transmission implicated in the 2022 outbreak. This has led to the establishment and spread of MPXV among the communities with high-risk sexual practices, especially with the anal receptive sex. Semen samples have been reported to harbor a high load of MPXV [5]. Human immunodeficiency virus (HIV) is another viral infection that is transmitted majorly through a sexual route [6]. The burden of HIV is high, with prevalent cases of 39 million and 630,000 deaths in 2022 alone. Within these cases, Africa is the worst affected, with 2/3 of the people living with HIV (PLHIV) on the continent [7]. Mpox is also endemic in African countries [8].

Considering the overlap in the epidemiology of HIV and mpox in the context of the 2022 multicountry mpox outbreak, it would be relevant to explore the relationship between these viral infections at the global and regional levels. The clinical manifestation of mpox is self-limiting, but the disease can be severe in a specific population, such as individuals with immunosuppressed, including PLHIV[9]. Studies have reported poor outcomes among individuals with MPXV -HIV co-infection [10,11,12,13]. In a retrospective study from Nigeria among 40 individuals with mpox, it was reported that individuals with MPXV-HIV were susceptible to an increase in the duration of the disease, more prominent lesions, and increased secondary bacterial infection [13]. Uncontrolled HIV is associated with adverse outcomes, including deaths [12].

The epidemiological profile of mpox has shown considerable variation between continents, endemic/nonendemic countries, and the period of the cases (2022 multicountry outbreak vs. Pre-2022) in terms of mortality rates, clinical manifestations, and demographic characteristics of the affected population [14,15,16,17]. Studies in Africa before the current outbreak reported a prevalence of HIV among mpox in the range of 18.75% to 25%[18,19]. Studies in European countries showed varied HIV prevalence ranging from 15% in France to 64% in Spain [20,21]. Thus, multiple studies from current and past outbreaks have reported varying HIV prevalence among individuals with mpox. However, based on our systematic search in multiple databases, we did not find an analysis reporting the pooled prevalence of HIV among the individuals with mpox, and the regional and temporal variations.

Hence, we conducted a systematic review and meta-analysis to determine the pooled prevalence of HIV reported among individuals with mpox, and to synthesise the epidemiological characteristics of individuals with HIV-MPXV co-infections.

## 2. Materials and Methods

### 2.1. Research Question & Selection Criteria

The present systematic review and meta-analysis were carried out based on the research question, ‘What is the prevalence of HIV in individuals with mpox?**’** The systematic search and identification of eligible studies were centered on the criteria elaborated in the Appendix A. (Table A1 and Table A2). The study population included laboratory-confirmed individuals with mpox, belonging to all age groups and sex. All suspected or probable individuals with mpox were excluded. The outcome of interest was the prevalence of HIV. All eligible human studies published in English that satisfied the above criteria were included in the analysis. The present meta-analysis was registered with the International Prospective Register of Systematic Reviews (PROSPERO), with reference ID CRD42022383275.

### 2.2. Databases included and Search Strategy

The search was carried out in seven databases: “PubMed, Scopus, Web of Science, EMBASE, ProQuest, EBSCOHost, and Cochrane”, until 4 January 2023 (Table A2). We also searched the pre-print servers such as “medRxiv, arXiv, bioRxiv, BioRN, ChiRxiv, ChiRN, and SSRN”. Furthermore, studies obtained by hand search in the references of eligible primary research papers and reviews, which met our eligibility criteria, were also included in the data extraction. The search keywords included “mpox” ‘MPXV’, ‘monkeypox’, ‘*AIDS*’, ‘HIV’ and ‘PLHA’. The database-wise search strategy applied and the results obtained have been enumerated in Table A2. The Mendeley Desktop V1.19.5 software was used to import articles, manage the citations, remove duplicates, and coordinate the overall review process between authors.

### 2.3. Selection of Studies

#### 2.3.1. Title-Abstract Screening

Four senior authors independently reviewed the title abstracts of the studies obtained from the above systematic search applying the eligibility criteria and identified articles for full-text screening. If there was a disagreement about including a study for full-text review, the co-authors conversed among themselves to build consensus and decided on eligibility.

#### 2.3.2. Full Text Screening & Data Extraction

**Figure 1:**
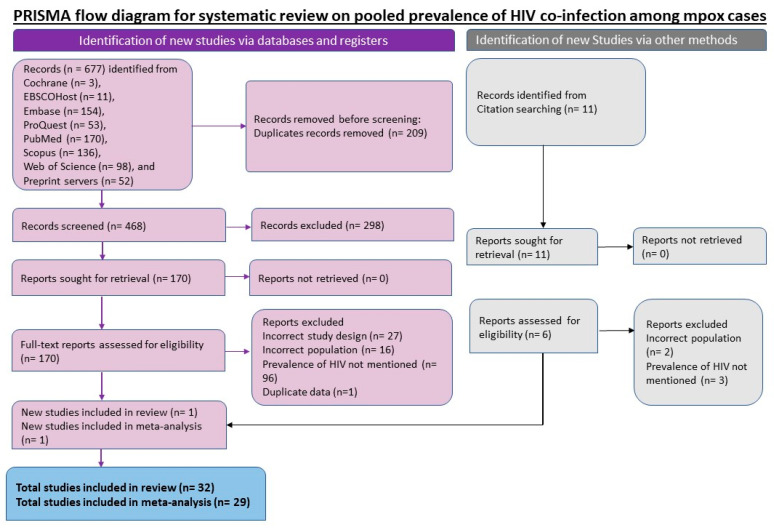
PRISMA flowchart for included studies in systematic review and meta-analysis of prevalence of HIV among the individuals with mpox.

Eligible full-text articles were reviewed for suitability of data extraction by two authors, and data extraction was performed by them independently. Contradictions in the extraction of data between the authors were removed in a consensus meeting held at the end of the independent extraction. A final table was formulated that included information such as author’s name, publication year, sex distribution, age, study where it was conducted, study design, total mpox-positive individuals, and PLHIV. The preferred reporting standard of systematic reviews and Meta-analysis (PRISMA) flow chart and checklist were utilised to report general search, screening, data extraction, systematic review, and meta-analysis conducted to ensure scientific precision (Figure 1 & Table A3).

### 2.4. Quality Assessment

The risk of bias in the included studies was independently evaluated by two authors (MAS&SM) using the National Heart, Lung, and Blood Institute (NHLBI) quality assessment tool for case series and cross-sectional studies [22].

### 2.5. Data Analysis

The pooled prevalence of HIV infection was estimated as a proportion by collating the total number of individuals with mpox and PLHIV. To address the risk of bias, we planned a sensitivity analysis by excluding poor-quality studies. Baujat plot, Leave-one-out analysis, radial plot, and diagnostic test were applied to identify the outliers and undertake sensitivity analysis. The I^2^ test was applied to determine heterogeneity among the included studies. Depending on the I^2^ value, heterogeneity can be declared low (25%), moderate (25–50%), and high (>50%). A random effect regression model with the DerSimonian & Laird estimator was used to evaluate the overall effect, as heterogeneity was high. We performed a subgroup analysis to identify the source of heterogeneity: (i) geography (continent-wise), (ii) MPXV (endemic vs. non endemic countries), and (iii) 2022 vs. pre-2022 studies. Prediction interval (PI) for the outcome was calculated if the heterogeneity was high. Meta-regression was performed to assess the effect of age and sex on the outcomes using bubble plots. The funnel plot and doi plot were used to evaluate publication bias. If the funnel plot depicted possible publication bias, a trim-and-fill test was planned to address publication bias, and the Eggers test to evaluate the effects of the small study was planned. A *p*-value of less than 0.05 was interpreted as statistically significant. All analyses and graphs were performed using R-programming (v4.0).

## 3. Results

### 3.1. Eligible Studies

Figure 1 shows the article selection process as PRISMA flow chart. The systematic search yielded 468 articles after removing 209 duplicates. After the title abstract selection, 170 articles were eligible for full-text selection. In the full-text screening, 139 articles were excluded due to non-reporting of HIV status (96), incorrect study design (27), and incorrect patient population (16). Two new studies were included from the hand-search of the references from the included studies. Finally, 32 studies were found eligible for systematic review. Among them, two studies included individuals with MPXV-HIV co-infection as their study population, and one study had a data duplication. Hence 29 studies were included for data extraction in the meta-analysis.

### 3.2. Study Characteristics

**Figure 2:**
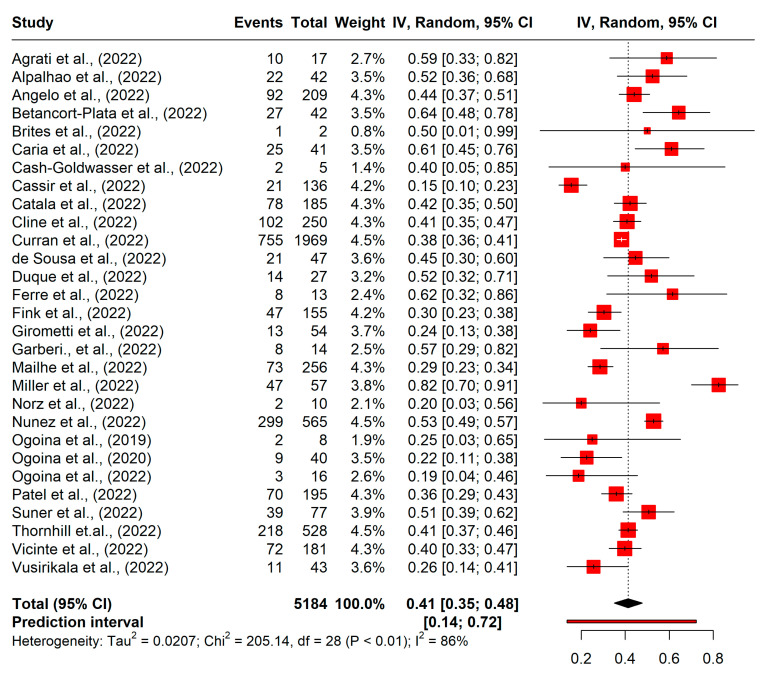
Forest plot of pooled prevalence of HIV prevalence in monkeypox virus.

The included studies were conducted from 1987 to 2022. Among the 29 studies included, seven were cross-sectional studies [11,18,19,20,23,24,25] seven were retrospective studies[13,21,26,27,28,29,30], seven were prospective studies[31,32,33,34,35,36,37], and eight were case series[38,39,40,41,42,43,44,45]. The studies had sample sizes ranging from 2 to 1969. The median age of the participants ranged from 16 to 42 years in all studies. The proportion of men among the mpox cases was 75% in all studies, with 14 studies reporting 100% men cases. The studies from the United States of America (USA) and Spain were the most common, that is, six studies each of 32 studies (18.75%), followed by Nigeria (4/32), Portugal (4/32), and the United Kingdom (4/32) (Table 1). Sexual practices of the individuals with mpox were reported in 82.76% (24/29) of the studies and 75.86% (22/29) studies reported on high-risk sexual behaviours and/or high risk groups (HRGs) [mostly men who have sex with men (MSM)]. Penile-anal (81%), followed by oral-penile and oral-anal, were the common modalities of sexual behaviour reported by these HRGs [23]. The highest prevalence of HIV infection (82.46%) was reported by a study in the USA [44], while the lowest prevalence was reported in France (15.44%).[46] The heterogeneity between the studies was assessed to be high (I^2^ = 86%; *p* < 0.001) (Figure 2). Therefore, the random-effects model was applied to determine the pooled prevalence.

### 3.3. Pooled Prevalence

The meta-analysis included a total of 5184 confirmed individuals with mpox, among whom 2091 reported HIV infection. The pooled prevalence of HIV infection in the target population was 41% (95% confidence interval [CI], 35–48) (Figure 2). The prediction interval was 14–71%.

### 3.4. Risk of Bias

The quality assessment of the findings of the included study is illustrated in the supplement file (Table A4), with all studies (30) rated as fair or good quality. The Doi plot was symmetrical with an LFK index of 0.91, indicating no publication bias (Figure S1). The layout of the funnel plot also showed a symmetric funnel, indicating no publication bias (Figure S2).

### 3.5. Sensitivity Analysis

**Figure 3:**
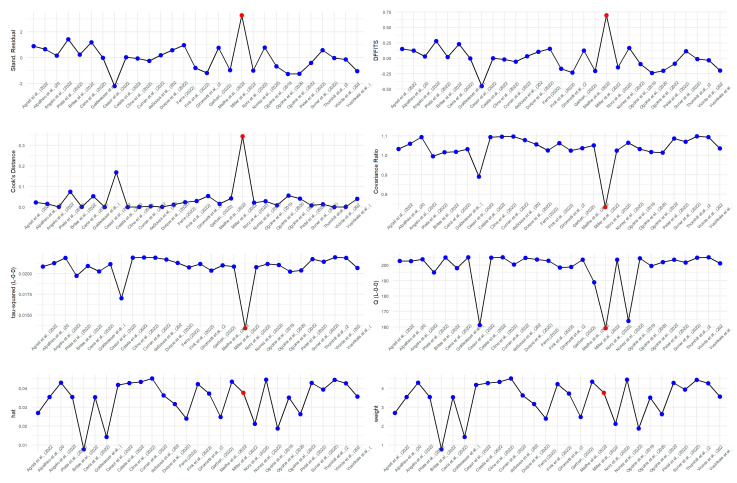
Sensitivity analysis: outlier detection by influence diagnostics.

**Figure 4:**
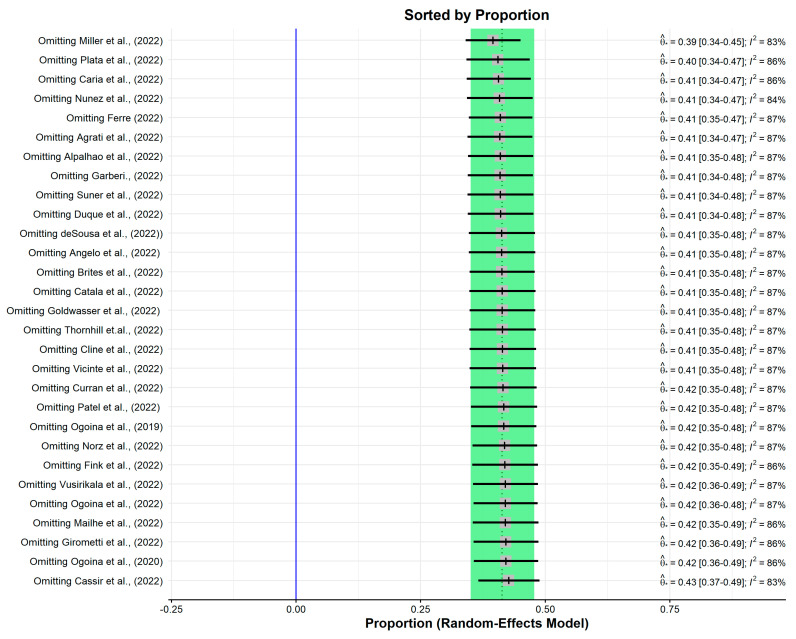
Sensitivity analysis by leave-one-out analysis.

Sensitivity analysis enables the improvement of the robustness of the pooled estimates by eliminating or reducing the heterogeneity between the studies. In order to undertake sensitivity analysis, diagnostic tests were applied [46]. One of the studies (Miller et al. [44]) was identified as an outlier by the diagnostic test (Figure 3). However, leave-one-out analysis did not yield a significant change in the pooled estimate or heterogeneity (Figure 4).

### 3.6. Subgroup Analysis

Based on the geography in which the study was conducted, African studies reported a relatively low prevalence of HIV infection of 21% (95% CI 15–28) among individuals with mpox, while studies from Europe reported 41% (95% CI 33–49). Studies in North America reported a higher prevalence of 52% (95% CI 28–76) (Figure S3). The prevalence of HIV infection has been higher during the ongoing multicountry outbreak [42% (95% CI 36–49)] than from the cases reported before the 2022 outbreak [22% (95% CI 4–47)] (Figure S4). Similarly, the prevalence of HIV infection in individuals with mpox differed significantly according to mpox endemicity, with a higher pooled prevalence among nonendemic countries [43% (95% CI 36–50)] than in endemic countries [21% (95% CI 15–28)] (Figure S5). However, subgrouping based on the geography (continent), endemicity, and time of occurrence of the cases did not reduce the heterogeneity.

The bubble graph showed that age had an inverse relationship and that the proportion of males in the study had a directly proportional relationship with HIV prevalence among individuals with mpox. (Figures S6 and S7) However, the relationships were not significant. Similarly, metaregression did not show a significant effect of sample size on HIV prevalence. (Figure S8).

## 4. Discussion

Our review and meta-analysis revealed a pooled prevalence of HIV infection of 41.11% among individuals with mpox. However, the prevalence varied according to the cases’ geography, endemicity, and period. Studies in an African country (Nigeria) and before 2022 reported a lower HIV prevalence of 21.22% and 22.05%, respectively. The significant difference in the HIV prevalence might be due to the predominant transmission mode during the pre-2022 multicountry outbreak, which was mainly through close contact with the skin lesions [5,18]. The ongoing multicountry outbreak and nonendemic countries were found to have a higher prevalence of HIV infection among individuals with mpox (42.05% and 42.60%, respectively). The prevalence rate is very high compared to the prevalence of HIV among the general population in all included countries (African and non-African countries). However, the gap was more pronounced in nonendemic and non-African countries, with a study from Portugal reporting a difference of more than 100 times that of the general population [38]. The shift in human-human transmission routes might explain this. The current outbreak, which is more concentrated in nonendemic European and American countries, has a disproportionate share of the MSM, bisexuals, and gay population [30], who are HRGs for HIV. Although the burden and prevalence are high in African countries, the proportion of HIV infection among individuals with mpox is relatively lower than in Europe and the Americas, where HIV prevalence is low. In contrast, mpox lesions can also aid and abate the transmission of sexually transmitted diseases, including HIV [38].

Mpox lesions in the primary areas of the body of sexual contacts have been suspected to be the transmission gateway in patients with high-risk behavior [24]. Rectal and semen samples had shown 67–77% positivity for individuals with mpox. A series of cases with MPXV-HIV coinfection reported the presence of the MPXV in 90% of the rectal swabs [47]. Although the viral load was lower, the median clearance of the MPXV from semen samples has been reported to be 13 days, extending up to 39 days among the majority of the patients [36]. Asymptomatic individuals with MSM mpox from France had their samples tested positive for MPXV from anal swabs, indicating a possible shedding of the virus in the anal route [28]. These might be a potential explanation for MPXV transmission among people with high-risk sexual behaviors.

Clinical characteristics varied according to HIV infection status, with MPXV-HIV coinfected individuals having significantly higher composite rash scores. A significantly higher rate of oral and perioral lesions was found among the PLHIVs [23]. Acute coinfection with HIV and MPXV has been reported to lead to hospitalization [27]. In a global case series, the hospitalization rate among the MPXV-HIV coinfected individuals was reported to be 28% [48]. Severity varied according to the status of HIV control, with adverse outcomes reported in hospitalized individuals with poor HIV control [44]. A study from Spain reported the most severe mpox infection in the PLHIV with poor disease control (CD4 = 265), than in the other individuals with HIV-MPXV [32]. Studies from nonendemic countries reported good HIV control status among most individuals with HIV- MPXV coinfection [13,28,30,31,35,45,49]. A study from mpox endemic Nigeria reported a low CD4 count among the individuals with MPXV-HIV coinfection [18]. A study from the USA reported that poorly controlled HIV status had a longer duration of hospitalization than HIV-negative individuals with mpox [40]. A study in Nigeria reported poor clinical results among MPXV-HIV coinfected individuals but did not report the control status of HIV [11]. Individuals with asymptomatic mpox who were found to have HIV coinfection (61.54%) had it under good control [28]. It was also found that, irrespective of HIV infection, individuals with mpox were able to elicit a T cell response against the mpox virus [20,31].

We also found that, during the review, all African studies reporting HIV status in individuals with mpox were from Nigeria. However, none from the Democratic Republic of the Congo (DRC) reported a HIV status. This is of great importance because, while Nigeria had clade II MPXV, the mpox cases in the DRC were due to the more severe and fatal clade (Clade I). Additionally, the current multicountry outbreak in the endemic countries is primarily driven by clade IIb, thus limiting information on the interaction of HIV with the more severe clade of mpox virus (Clade I). A better understanding of the MPXV-HIV coinfection in the endemic African context, especially in the DRC, requires all future studies to report the HIV status of individuals with mpox.

### Strengths and Limitations

This review included studies in multiple databases of published and unpublished literature to quantify and study the prevalence of HIV in mpox patients, globally and regionally. The risk of bias in the studies was assessed using standard tools. Subgroup analysis and metaregression (including multiple variables, such as sex, age, and sample size) were undertaken to explain the heterogeneity. A sensitivity analysis was performed methodologically, identifying and excluding outliers. However, the present analysis was not without limitations. There was significantly high heterogeneity among the included studies. The substantial heterogeneity observed in the study is hypothesized due to the global nature of the data in the review with varied patient characteristics in terms of geographic location, high-risk behavior, the types of HIV (HIV-1 and HIV-2), clades and lineages of the MPXV, and other comorbidities in the various studies.

## 5. Conclusions

Overall, the pooled prevalence of HIV infection among individuals with mpox was 41%. A relatively lower prevalence of HIV was observed in Africa, whereas a higher prevalence of HIV was found among nonendemic countries. Considering the high prevalence of HIV among individuals with mpox and the adverse outcomes reported among individuals with uncontrolled HIV, HIV testing for all persons with unknown HIV status may be conducted under routine investigation. A history of sexual orientation and of sexual partners in the last 21 days may be taken from individuals with mpox to identify the potential source and contacts for quarantining and testing them as part of the public health response. More epidemiological and analytical studies must be conducted to explore the relationship between HIV infection and mpox and the mediating role of high-risk sexual practices.

## Figures and Tables

**Table 1:** Baseline characteristics of studies reporting proportion of HIV infection in individuals with mpox.

Author (Year)	Study Design	Country	Total Cases	HIV Positive	HIV Co-Infection Prevalence	Proportion of Male Mpox Cases	Median Age (Years)	Key Findings
Agrati et al. (2022) [31]	Prospective observational study	Italy	17	10	58.82%	100.0	40	Among 17 individuals with mpox, 10 were reported as HIV positive.
Alpalhao et al. (2022) [38]	Retrospective Cohort Study	Portugal	42	22	52.38%	100.0	-	Among the 42 individuals with mpox, 22 were HIV positive.
Angelo et al. (2022) [23]	Cross-sectional	15 countries	209	92	44.02%	100.0	37	Data was available only on the 211 patients. Of the 209 patients, 92 were HIV positive.
Betancort-Plata et al. (2022) [20]	Cross-sectional	Spain	42	27	64.29%	100.0	16	HIV-positive status was found in 27 patients.
Brites et al. (2022) [39]	Cross-sectional	Brazil	2	1	50.00%	100.0	34	Of these two cases 1 patient living with HIV infection.
Caria et al. (2022) [26]	Retrospective Cohort Study	Portugal	41	25	60.98%	97.6	37	Of the total 4117 individuals with mpox, 25 individuals were HIV positive.
Cash-goldwasser et al. (2022) [40]	Cross-sectional	USA	5	2	40.00%	80.0	-	A total of 517 individuals with mpox were considered in the study and 2 patients were HIV positive.
Cassir et al. (2022) [21]	Retrospective Cohort Study	France	136	21	15.44%	97.8	36	The study included 136 laboratory-confirmed mpox patients. Among 136, 21 patients were HIV positive.
Catala et al. (2022) [24]	Prospective observational study	Spain	185	78	42.16%	100.0	-	A total of 185 patients were included in the study. HIV infection was seen in 78 patients.
Cline et al. (2022) [25]	Cross-sectional	USA	250	102	40.80%	96.8	-	A total of 250 laboratory-confirmed patients were included and 41% of patients were HIV positive.
Curran et al. (2022) [11]	Cross-sectional	USA	1969	755	38.34%	99.3	35	Of the total 1969 patients, 755 were HIV positive.
de Sousa et al. (2022) [27]	Retrospective Cohort Study	Portugal	47	21	44.68%	100.0	-	A total of 47 patients were included in the study. Of the total patients, 44.7% of the patients were HIV positive.
Duque M et al. (2022) [41]	Case series	Portugal	27	14	51.85%	100.0	33	A total of 14 out of 27 mpox cases were HIV positive (51.85%).
Ferré (2022) [28]	Retrospective Cohort Study	France	13	8	61.54%	100.0	38	Monkeypox was positive in 13 patients and 8 patients were living with HIV.
Fink et al. (2022) [29]	Retrospective Cohort Study	United Kingdom	155	47	30.32%	98.1	35	The study included 156 monkeypox patients. Among 155 patients, HIV was positive in 30%.
Girometti et al. (2022) [30]	Retrospective Cohort Study	United Kingdom	54	13	24.07%	100.0	41	The study includes 54 monkeypox-diagnosed patients and 24% (13) of people living with HIV were identified.
Gomez-Garberi et al (2022) [32]	Prospective observational study	Spain	14	8	57.14%	100.0	42	The study recruited a total of 14 patients. HIV was found in 8 patients.
Mailhe M et al. (2022) [33]	Prospective observational study	France	256	73	28.52%	99.2	35	Of the total 256 mpox patients, 73 were found to be HIV positive (28.5%).
Miller MJ et al. (2022) [44]	Case series	USA	57	47	82.46%	94.7	34	Out of 57 monkeypox cases, 47 (82.4%) were HIV positive, and all 47 patients were taking ART.
Nörz D et al. (2022) [34]	Prospective observational study	Germany	10	2	20.00%	100.0	-	Two out of ten mpox cases two (20%) were found to be HIV positive, and both two were under ART.
Núñez I et al. (2022) [35]	Prospective observational study	Mexico	565	299	52.92%	97.2	36	Out of 565 patients, 299 patients (52.9%) were found to be HIV positive.
Ogoina D et al. (2019) [18]	Cross-sectional	Nigeria	8	2	25.00%		-	Laboratory investigation was performed among 8 patients and 2 were identified as HIV positive.
Ogoina D et al. (2020) [13]	Retrospective Cohort Study	Nigeria	40	9	22.50%		32	The study included 40 monkeypox cases. Of the 40 patients, 9 were HIV positive.
Ogoina D et al. (2022) [19]	Cross-sectional	Nigeria	16	3	18.75%		28	Out of 16 mpox cases, 3 cases were found to be HIV positive (18.75%).
Patel et al. (2022) [45]	Case series	United Kingdom	195	70	35.90%		38	Of the total 197 patients, 70 were HIV positive (35.5%).
Suñer et al. (2022) [36]	Prospective observational study	Spain	77	39	50.65%		35	A total of 77 monkeypox patients were included. HIV was positive in 51% of the patients.
Thornhill et.al., (2022) [43]	Case series	America, Europe, Israel, or Australia.	528	218	41.29%		38	A total of 218 (41%) of the 528 patients had reported HIV infection.
Vicinte et al. (2022) [37]	Prospective observational study	Spain	181	72	39.78%		37	Of the 181 patients, 72(40%) had reported HIV infection.
Vivancos-Gallego MJ et al. (2022) [47]	Case series	Spain	25	25	100.00%		-	All 25 patients were HIV-diagnosed monkeypox cases; the mean age was 39.5 years.
Vusirikala A et al. (2022) [42]	Case series	United Kingdom	43	11	25.58%		40	A total of 11 out of 45 mpox cases were HIV positive (24%).

## Data Availability

Data are available in the studies used for this review.

## Supplementary Materials

The following supporting information can be downloaded at: https://www.mdpi.com/article/10.3390/epidemiologia4030033/s1, Figure S1: Doi plot of pooled prevalence of HIV among individuals with mpox. Figure S2: Funnel plot with a 95% confidence interval of pooled prevalence of HIV among individuals with mpox. Figure S3: Forest plot of the subgroup analysis according to the place of the study. Figure S4: Forest plot of the su-group analysis according to the period of study. Figure S5: Forest plot of the subgroup analysis according to endemicity of mpox. Figure S6: Bubble plot demonstrating metaregression based upon the participant’s age. Figure S7: Bubble plot demonstrating metaregression based upon the proportion of male cases. Figure S8: Bubble plot demonstrating metaregression based upon sample size.

## Appendix A

**Table A1:** Inclusion and exclusion criteria.

Research Question: What Is the Prevalence of HIV Among Individuals with Mpox (Monkeypox) Patients?		
Inclusion		Exclusion
**Participants**	Laboratory-confirmed individuals with mpox All gender All age groups	Suspected or Probable individuals with mpox
**Disease**	HIV (self-reported or documented)	
**Outcome**	Prevalence of HIV	Risk factors for HIV
	Subgroup analysis according to gender	Outcomes and Mortality studies
**Study Designs**	Prevalence studies, case series, cross-sectional studies, cohort studies, case-control studies, and surveys	Qualitative, policy, opinion, case studies, case-reports
	Geography-global level Date of Search-Published till 4 January 2023 English Language Human studies	
	Published and unpublished data	

**Table A2:** The adjusted search terms as per searched electronic databases [as of 04.01.2023].

Database	No	Search Query	Results
Cochrane			
	#1	(mpox:ti,ab) OR (monkeypox:ti,ab) OR (mpxv:ti,ab) OR (“monkey pox”:ti,ab)	14
	#2	“human immunodeficiency virus”:ti,ab OR “human t cell lymphotropic virus type iii”:ti,ab OR “lymphotropic virus type iii”:ti,ab OR (“aids” NEXT virus*):ti,ab OR “acquired immune deficiency syndrome virus”:ti,ab OR “acquired immunodeficiency syndrome virus”:ti,ab OR “acquired immune deficiency syndrome”:ti,ab OR “acquired immuno deficiency syndrome”:ti,ab OR AIDS:ti,ab OR “people living with hiv aids”:ti,ab OR PLHA:ti,ab OR HIV:ti,ab	33,277
	#3	#1 AND #2	3
EBSCOHost-Academic Search Complete			
	#1	((TI mpox OR AB mpox)) OR ((TI monkeypox OR AB monkeypox)) OR ((TI mpxv OR AB mpxv)) OR ((TI “monkey pox” OR AB “monkey pox”))	103
	#2	(TI “human immunodeficiency virus” OR AB “human immunodeficiency virus”) OR (TI “human t cell lymphotropic virus type iii” OR AB “human t cell lymphotropic virus type iii”) OR (TI “lymphotropic virus type iii” OR AB “lymphotropic virus type iii”) OR (TI “aids virus*”OR AB “aids virus*”) OR (TI “acquired immune deficiency syndrome virus” OR AB “acquired immune deficiency syndrome virus”) OR (TI “acquired immunodeficiency syndrome virus” OR AB “acquired immunodeficiency syndrome virus”) OR (TI “acquired immune deficiency syndrome” OR AB “acquired immune deficiency syndrome”) OR (TI “acquired immuno deficiency syndrome” OR AB “acquired immuno deficiency syndrome”) OR (TI AIDS OR AB AIDS) OR (TI “people living with hiv aids” OR AB “people living with hiv aids”) OR (TI PLHA OR AB PLHA) OR (TI HIV OR AB HIV)	27,692
	#3	#1 AND #2	11
EMBASE			
	#1	(mpox:ti,ab) OR (monkeypox:ti,ab) OR (mpxv:ti,ab) OR (‘monkeypox’:ti,ab)	2581
	#2	‘human immunodeficiency virus’:ti,ab OR ‘human t cell lymphotropic virus type iii’:ti,ab OR ‘lymphotropic virus type iii’:ti,ab OR ‘aids virus*’:ti,ab OR ‘acquired immune deficiency syndrome virus’:ti,ab OR ‘acquired immunodeficiency syndrome virus’:ti,ab OR ‘acquired immune deficiency syndrome’:ti,ab OR ‘acquired immuno deficiency syndrome’:ti,ab OR AIDS:ti,ab OR ‘people living with hiv aids’:ti,ab OR PLHA:ti,ab OR HIV:ti,ab	566,993
	#3	#1 AND #2	154
ProQuest			
	#1	(TI,AB(mpox)) OR (TI,AB(monkeypox)) OR (TI,AB(mpxv)) OR (TI,AB(“monkey pox”))	555
	#2	TI,AB(“human immunodeficiency virus”) OR TI,AB(“human t cell lymphotropic virus type iii”) OR TI,AB(“lymphotropic virus type iii”)OR TI,AB(“aids virus*”) OR TI,AB(“acquired immune deficiency syndrome virus”) OR TI,AB(“acquired immunodeficiency syndrome virus”) OR TI,AB(“acquired immune deficiency syndrome”) OR TI,AB(“acquired immuno deficiency syndrome”) OR TI,AB(AIDS) OR TI,AB(“people living with hiv aids”) OR TI,AB(PLHA) OR TI,AB(HIV)	211,085
	#3	1 AND 2	53
PubMed			
	#1	(mpox[Title/Abstract]) OR (monkeypox[Title/Abstract]) OR (mpxv[Title/Abstract]) OR (“monkey pox”[Title/Abstract]) OR (“monkeypox”[MeSH Terms]) OR (“monkeypox virus”[MeSH Terms])	2441
	#2	“human immunodeficiency virus”[Title/Abstract] OR “human t cell lymphotropic virus type iii”[Title/Abstract] OR “lymphotropic virus type iii”[Title/Abstract] OR “aids virus*”[Title/Abstract] OR “acquired immune deficiency syndrome virus”[Title/Abstract] OR “acquired immunodeficiency syndrome virus”[Title/Abstract] OR “acquired immune deficiency syndrome”[Title/Abstract] OR “acquired immuno deficiency syndrome”[Title/Abstract] OR “AIDS”[Title/Abstract] OR “people living with hiv aids”[Title/Abstract] OR “PLHA”[Title/Abstract] OR “HIV”[Title/Abstract] OR “HIV”[MeSH Terms] OR “acquired immunodeficiency syndrome”[MeSH Terms]	466,547
	#3	1 AND 2	170
Scopus			
	#1	(TITLE-ABS(mpox)) OR (TITLE-ABS(monkeypox)) OR (TITLE-ABS(mpxv)) OR (TITLE-ABS(“monkey pox”))	2397
	#2	TITLE-ABS(“human immunodeficiency virus”) OR TITLE-ABS(“human t cell lymphotropic virus type iii”) OR TITLE-ABS(“lymphotropic virus type iii”) OR TITLE-ABS(“aids virus*”) OR TITLE-ABS(“acquired immune deficiency syndrome virus”) OR TITLE-ABS(“acquired immunodeficiency syndrome virus”) OR TITLE-ABS(“acquired immune deficiency syndrome”) OR TITLE-ABS(“acquired immuno deficiency syndrome”) OR TITLE-ABS(AIDS) OR TITLE-ABS(“people living with hiv aids”) OR TITLE-ABS(PLHA) OR TITLE-ABS(HIV)	569,725
	#3	#1 AND #2	136
Web of Science			
	#1	((TI = mpox OR AB = mpox)) OR ((TI = monkeypox OR AB = monkeypox)) OR ((TI = mpxv OR AB = mpxv))	1660
	#2	(TI = AIDS OR AB = AIDS) OR (TI = PLHA OR AB = PLHA) OR (TI = HIV OR AB = HIV)	710,768
	#3	#1 AND #2	98

**Table A3:** PRISMA Checklist.

Section and Topic	Item #	Checklist Item	Location Where Item Is Reported
Title			
Title	1	Identify the report as a systematic review.	1
Abstract			
Abstract	2	See the PRISMA 2020 for the abstract checklist (created as per the journal guidelines).	2,3
Introduction			
Rationale	3	Describe the rationale for the review in the context of existing knowledge.	3,4
Objectives	4	Provide an explicit statement of the objective(s) or question(s) the review addresses.	4
Methods			
Eligibility criteria	5	Specify the inclusion and exclusion criteria for the review and how studies were grouped for syntheses.	4 and Table A1
Information sources	6	Specify all databases, registers, websites, organizations, reference lists, and other sources searched or consulted to identify studies. Specify the date when each source was last searched or consulted.	4,5 and Table A2
Search strategy	7	Present the full search strategies for all databases, registers, and websites, including any filters and limits used.	Table A2
Selection process	8	Specify the methods used to decide whether a study met the inclusion criteria of the review, including how many reviewers screened each record and each report retrieved, whether they worked independently, and if applicable, include details of automation tools used in the process.	5
Data collection process	9	Specify the methods used to collect data from reports, including how many reviewers collected data from each report, whether they worked independently, any processes for obtaining or confirming data from study investigators, and if applicable, include details of automation tools used in the process.	5
Data items	10a	List and define all outcomes for which data were sought. Specify whether all results that were compatible with each outcome domain in each study were sought (e.g., for all measures, time points, and analyses), and if not, the methods used to decide which results to collect.	5, Table 1
	10b	List and define all other variables for which data were sought (e.g., participant and intervention characteristics, funding sources). Describe any assumptions made about any missing or unclear information.	5, Table 1
Study risk of bias assessment	11	Specify the methods used to assess the risk of bias in the included studies, including details of the tool(s) used, how many reviewers assessed each study and whether they worked independently, and if applicable, details of automation tools used in the process.	4, Table A4
Effect measures	12	Specify for each outcome the effect measure(s) (e.g., risk ratio, mean difference) used in the synthesis or presentation of results.	5
Synthesis methods	13a	Describe the processes used to decide which studies were eligible for each synthesis (e.g., tabulating the study intervention characteristics and comparing against the planned groups for each synthesis (item #5)).	5, Table 1
	13b	Describe any methods required to prepare the data for presentation or synthesis, such as handling of missing summary statistics, or data conversions.	NA
	13c	Describe any methods used to tabulate or visually display the results of individual studies and syntheses.	Figure 1
	13d	Describe any methods used to synthesize results and provide a rationale for the choice(s). If meta-analysis was performed, describe the model(s), method(s) to identify the presence and extent of statistical heterogeneity, and software package(s) used.	5,6
	13e	Describe any methods used to explore possible causes of heterogeneity among study results (e.g., subgroup analysis, metaregression).	6
	13f	Describe any sensitivity analyses conducted to assess the robustness of the synthesised results.	5
Reporting bias assessment	14	Describe any methods used to assess the risk of bias due to missing results in a synthesis (arising from reporting biases).	NA
Certainty assessment	15	Describe any methods used to assess certainty (or confidence) in the body of evidence for an outcome.	NA
Results			
Study selection	16a	Describe the results of the search and selection process, from the number of records identified in the search to the number of studies included in the review, ideally using a flow diagram.	6
	16b	Cite studies that might appear to meet the inclusion criteria but were excluded and explain why they were excluded.	Table A1
Study characteristics	17	Cite each included study and present its characteristics.	6,7
Risk of bias in studies	18	Present assessments of the risk of bias for each included study.	7, Table A4
Results of individual studies	19	For all outcomes, present, for each study: (a) summary statistics for each group (where appropriate) and (b) an effect estimate and its precision (e.g., confidence/credible interval), ideally using structured tables or plots.	Table 1
Results of syntheses	20a	For each synthesis, briefly summarize the characteristics and risk of bias among the contributing studies.	7
	20b	Present the results of all statistical syntheses conducted. If meta-analysis was performed, present for each the summary estimate and its precision (e.g., confidence/credible interval) and measures of statistical heterogeneity. If comparing groups, describe the direction of the effect.	7,8
	20c	Present the results of all investigations into possible causes of heterogeneity among study results.	7,8
	20d	Present the results of all sensitivity analyses conducted to assess the robustness of the synthesised results.	7, Figure 3
Reporting biases	21	Present assessments of the risk of bias due to missing results (arising from reporting biases) for each synthesis assessed.	NA
Certainty of evidence	22	Present assessments of certainty (or confidence) in the body of evidence for each outcome assessed.	NA
Discussion			
Discussion	23a	Provide a general interpretation of the results in the context of other evidence.	8,9
	23b	Discuss any limitations of the evidence included in the review.	10
	23c	Discuss any limitations of the review processes used.	10
	23d	Discuss the implications of the results for practice, policy, and future research.	10
Other Information			
Registration and protocol	24a	Provide registration information for the review, including registration name and registration number, or state that the review was not registered.	4
	24b	Indicate where the review protocol can be accessed or state that a protocol was not prepared.	4
	24c	Describe and explain any amendments to the information provided at registration or in the protocol.	NA
Support	25	Describe the sources of financial or nonfinancial support for the review and the role of the funders or sponsors in the review.	11
Competing interests	26	Declare any competing interests of the review authors.	11
Availability of data, code, and other materials	27	Report which of the following are publicly available and where they can be found: template data collection forms; data extracted from included studies; data used for all analyses; analytic code; any other materials used in the review.	11

From: Page MJ, McKenzie JE, Bossuyt PM, Boutron I, Hoffmann TC, Mulrow CD, et al. The PRISMA 2020 statement: an updated guideline for reporting systematic reviews. BMJ 2021; 372: n71. doi: 10.1136/bmj.n71.

**Table A4:** (a) Quality assessment of the included case series with the use of the NIH quality assessment tool. (b) Quality assessment of included cross-sectional and cohort studies with the use of the NIH quality assessment tool.

(a)															
**Author (YOP)**	Q1	Q2	Q3		Q4		Q5		Q6	Q7		Q8	Q9		Overall Quality
**Patel et al. (2022)**	Y	Y	Y		Y		NA		Y	NA		Y	Y		Good
**Vusirikala et al. (2022)**	N	Y	N		Y		NA		Y	NA		Y	Y		Fair
**Thornill et al. (2022)**	Y	Y	NI		N		NA		Y	NA		Y	Y		Fair
**Alpalhao et al. (2022)**	Y	Y	Y		Y		NA		Y	NA		Y	Y		Good
**Brites et al. (2022)**	Y	Y	Y		Y		NA		Y	NA		NA	Y		Good
**Cash-Goldwasser et al. (2022)**	Y	Y	NI		Y		NA		Y	NA		NA	Y		Good
**Duque et al. (2022)**	Y	Y	Y		Y		NA		Y	NA		N	Y		Good
**Miller et al. (2022)**	Y	Y	Y		Y		NA		Y	Y		N	Y		Good
(b)															
**Author (YOP)**	Q1	Q2	Q3	Q4	Q5	Q6	Q7	Q8	Q9	Q10	Q11	Q12	Q13	Q14	Quality rating
**Ogoina et al. (2019)**	Y	Y	Y	Y	N	NI	NA	NA	Y	NI	Y	N	Y	N	Fair
**Ogoina et al. (2020)**	Y	Y	Y	Y	N	NI	NA	NA	Y	NI	Y	N	Y	N	Fair
**Tarin Vicinte et al. (2022)**	Y	Y	Y	Y	N	NI	NA	NA	Y	NI	Y	N	Y	N	Fair
**Nunez et al. (2022)**	Y	Y	Y	Y	N	NI	NA	NA	Y	NI	Y	N	Y	N	Fair
**Ogoina et al. (2022)**	Y	Y	Y	Y	N	NI	NA	NA	Y	NI	Y	N	Y	N	Fair
**Suner et al. (2022)**	Y	Y	Y	Y	N	NI	NA	NA	Y	NI	Y	N	Y	N	Fair
**Agrati et al. (2022)**	Y	Y	Y	Y	N	NI	NA	NA	Y	NI	Y	N	Y	N	Fair
**Angelo et al. (2022)**	Y	Y	Y	Y	N	NI	NA	NA	Y	NI	Y	N	Y	N	Fair
**Plata et al. (2022)**	Y	Y	Y	Y	N	NI	NA	NA	Y	NI	Y	N	Y	N	Fair
**Caria et al. (2022)**	Y	Y	Y	Y	N	NI	NA	NA	Y	NA	Y	N	Y	N	Fair
**Cassir et al. (2022)**	Y	Y	Y	Y	N	NI	NA	NA	Y	NA	Y	N	Y	N	Fair
**Catala et al. (2022)**	Y	Y	Y	Y	N	NI	NA	NA	Y	NA	Y	N	Y	N	Fair
**Cline et al. (2022)**	Y	Y	Y	Y	N	NI	NA	NA	Y	NA	Y	N	Y	N	Fair
**Curran et al. (2022)**	Y	Y	Y	Y	N	NI	NA	NA	Y	NI	Y	N	Y	N	Fair
**deSousa et al. (2022)**	Y	Y	Y	Y	N	NI	NA	NA	Y	NI	Y	N	Y	N	Fair
**Fink et al. (2022)**	Y	Y	Y	Y	N	NI	NA	NA	Y	NI	Y	N	Y	Y	Fair
**Girometti et al. (2022)**	Y	Y	Y	Y	N	NI	NA	NA	Y	NI	Y	N	Y	N	Fair
**Garberi et al. (2022)**	Y	Y	Y	Y	N	NI	NA	NA	Y	NI	Y	N	Y	N	Fair
**Mailhe et al. (2022)**	Y	Y	Y	Y	N	NI	NA	NA	Y	NI	Y	N	Y	N	Fair
**Norz et al. (2022)**	Y	Y	Y	Y	N	NI	NA	NA	Y	NI	Y	N	Y	N	Fair
**Ferre et al. (2022)**	Y	Y	Y	Y	N	NI	NA	NA	Y	NI	Y	N	Y	N	Fair

(a) YOP: Year of Publication; Y: Yes; N: No; NA: Not Applicable; CD: Cannot Determine; NI: No Information; NIH: National Institute of Health. Q1: Was the study question or objective clearly stated? Q2: Was the study population clearly and fully described, including a case definition? Q3: Were the cases consecutive? Q4: Were the subjects comparable? Q5: Was the intervention clearly described? Q6: Were the outcome measures clearly defined, valid, reliable, and implemented consistently across all study participants? Q7: Was the length of the follow-up adequate? Q8: Were the statistical methods well described? Q9: Were the results well described? (b) YOP: Year of Publication; Y: Yes; N: No; NA: Not Applicable; CD: Cannot Determine; NI: No Information. Q1: Was the research question or objective in this paper clearly stated? Q2: Was the study population clearly specified and defined? Q3: Was the participation rate of eligible persons at least 50%? Q4: Were all the subjects selected or recruited from the same or similar populations (including the same time period)? Were inclusion and exclusion criteria for being in the study prespecified and applied uniformly to all participants? Q5: Was a sample size justification, power description, or variance and effect estimates provided? Q6: For the analyses in this paper, were the exposure(s) of interest measured prior to the outcome(s) being measured? Q7: Was the timeframe sufficient so that one could reasonably expect to see an association between exposure and outcome if it existed? Q8: For exposures that can vary in amount or level, did the study examine different levels of the exposure as related to the outcome (e.g., categories of exposure, or exposure measured as a continuous variable)? Q9: Were the exposure measures (independent variables) clearly defined, valid, reliable, and implemented consistently across all study participants? Q10: Was the exposure(s) assessed more than once over time? Q11: Were the outcome measures (dependent variables) clearly defined, valid, reliable, and implemented consistently across all study participants? Q12: Were the outcome assessors blinded to the exposure status of participants? Q13: Was the loss to follow-up after baseline 20% or less? Q14: Were key potential confounding variables measured and adjusted statistically for their impact on the relationship between exposure(s) and outcome(s)?
