# Supplementary material for: Monkeypox Patients Living with HIV: A Systematic Review and Meta-Analysis of Geographic and Temporal Variations
Source: Epidemiologia (Basel). 2023 Sep 4;4(3):352–69. doi: 10.3390/epidemiologia4030033 (PMC10528863; doi:10.3390/epidemiologia4030033)
Supplement: Supplementary file 1 [file epidemiologia-04-00033-s001.zip › epidemiologia-2431124-supplementary.pptx]

## Slide 1
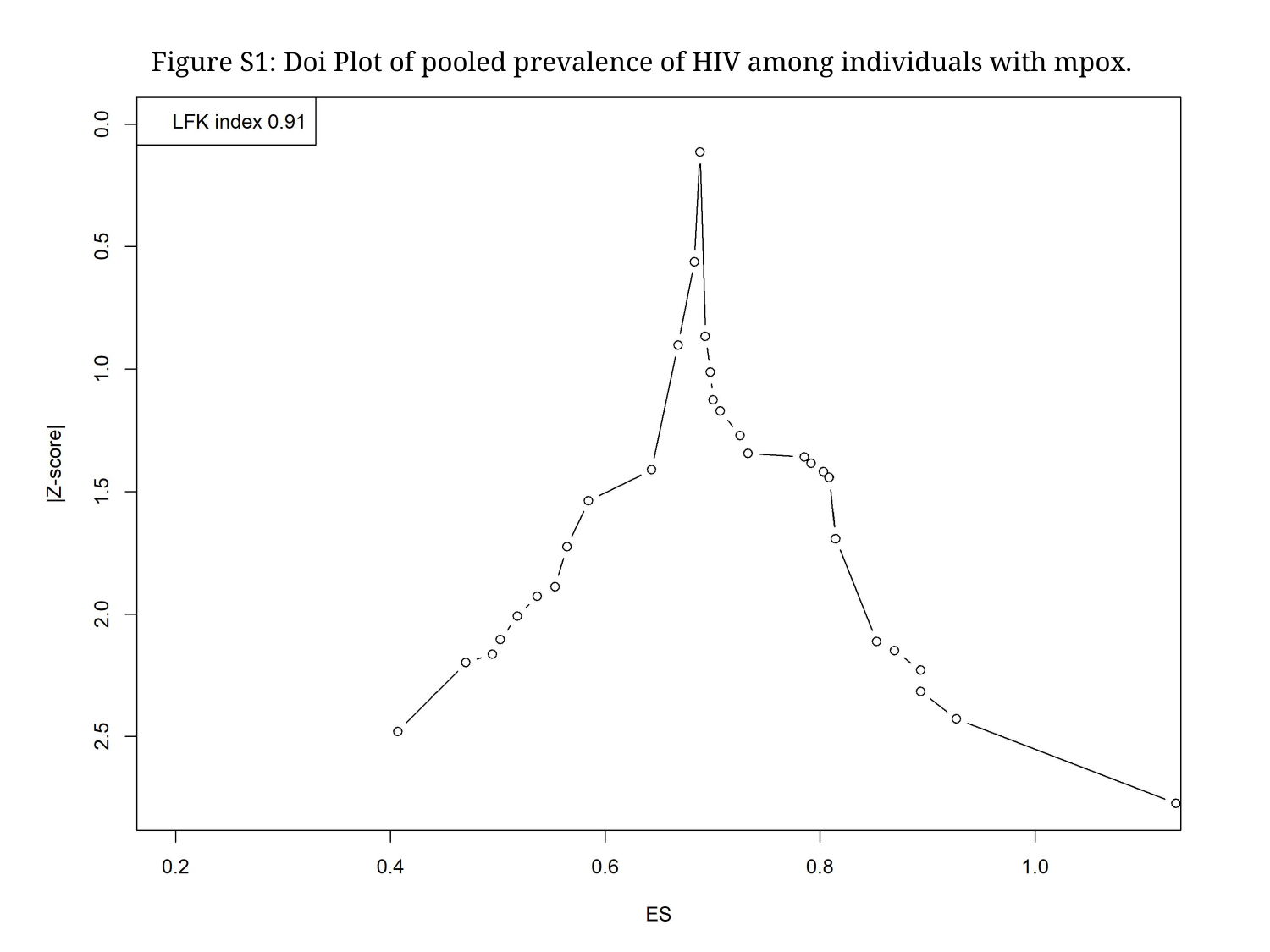

Figure S1: Doi Plot of pooled prevalence of HIV among individuals with mpox.

## Slide 2
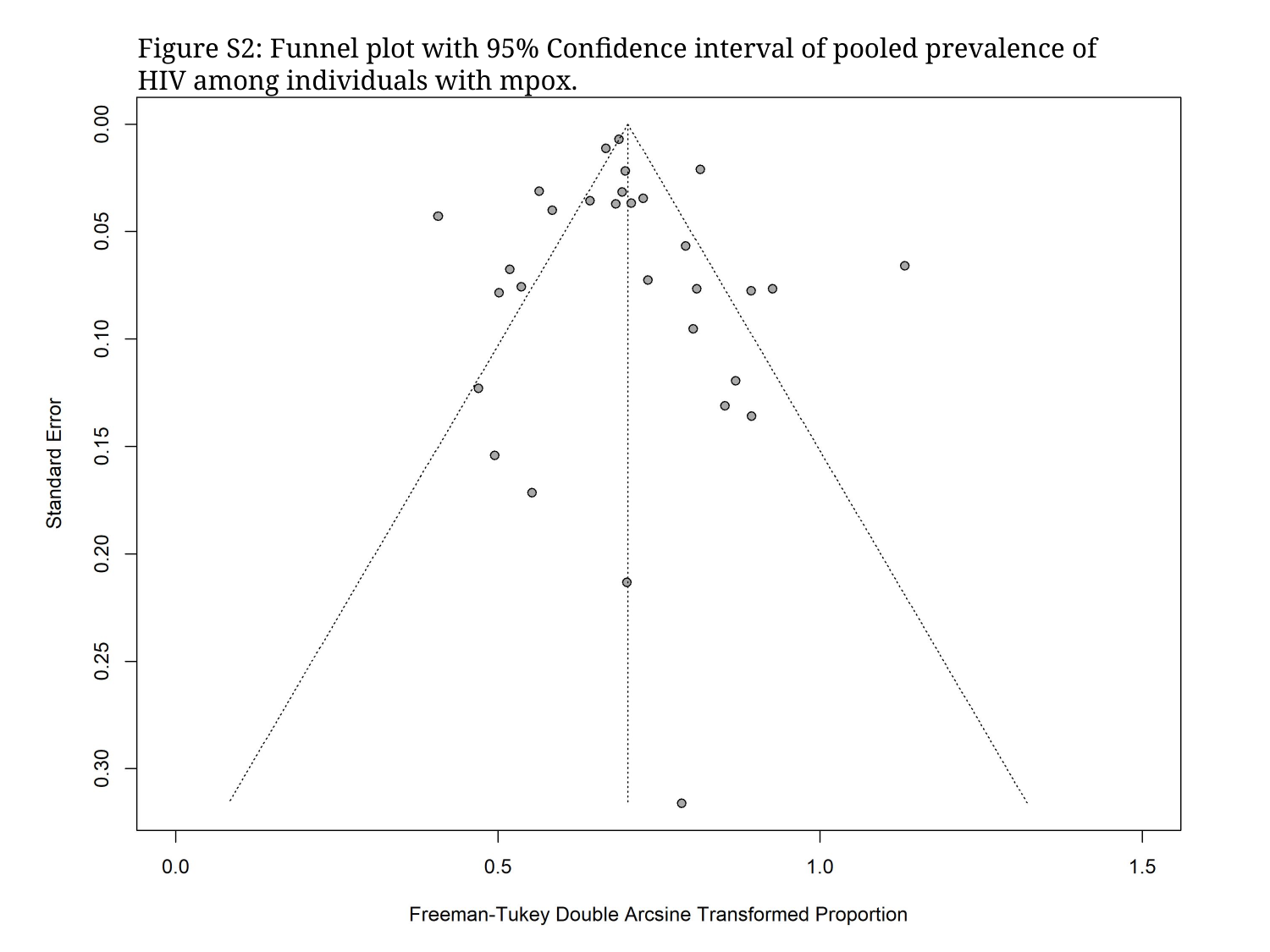

Figure S2: Funnel plot with 95% Confidence interval of pooled prevalence of HIV among individuals with mpox.

## Slide 3
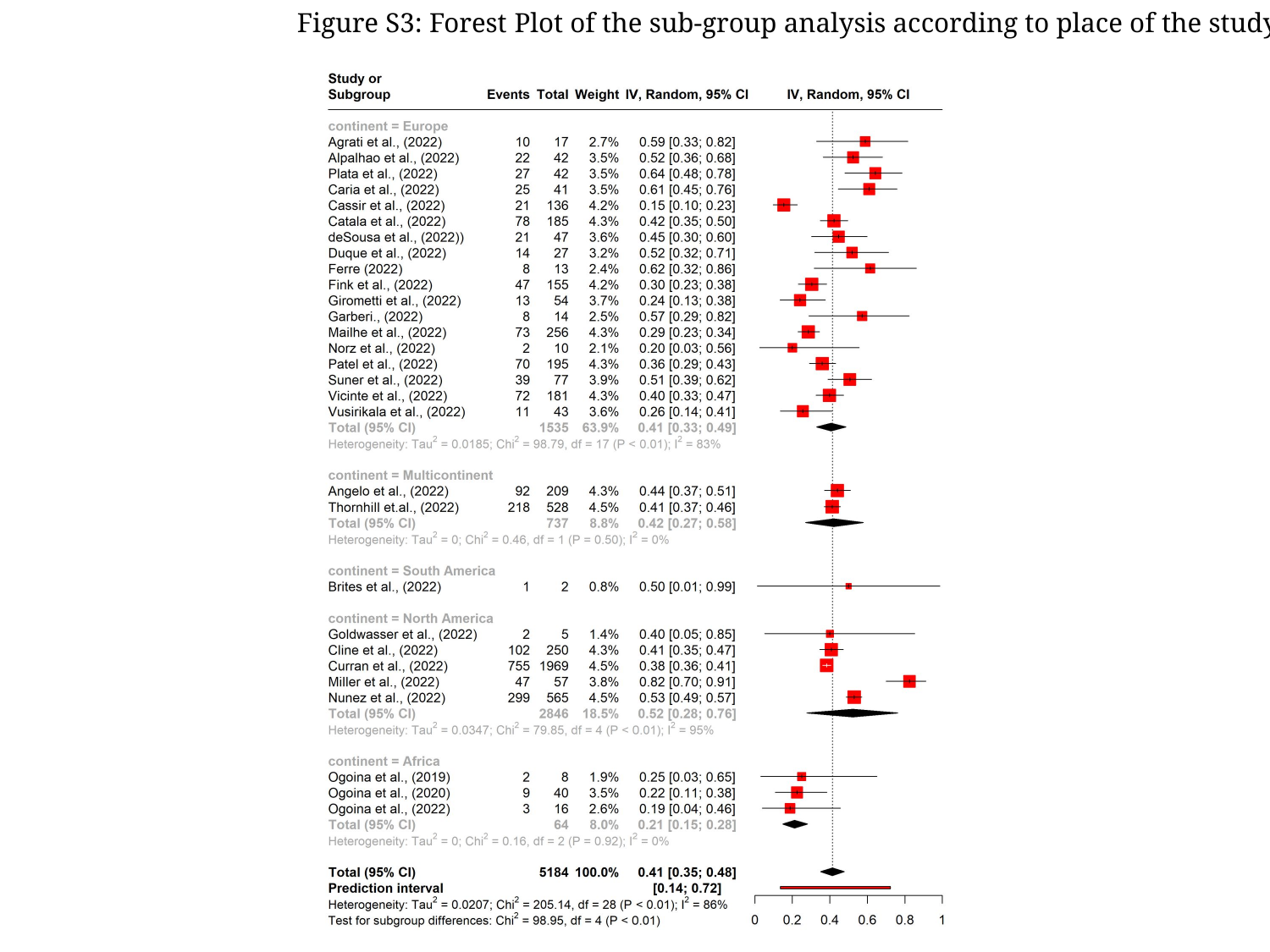

Figure S3: Forest Plot of the sub-group analysis according to place of the study

## Slide 4
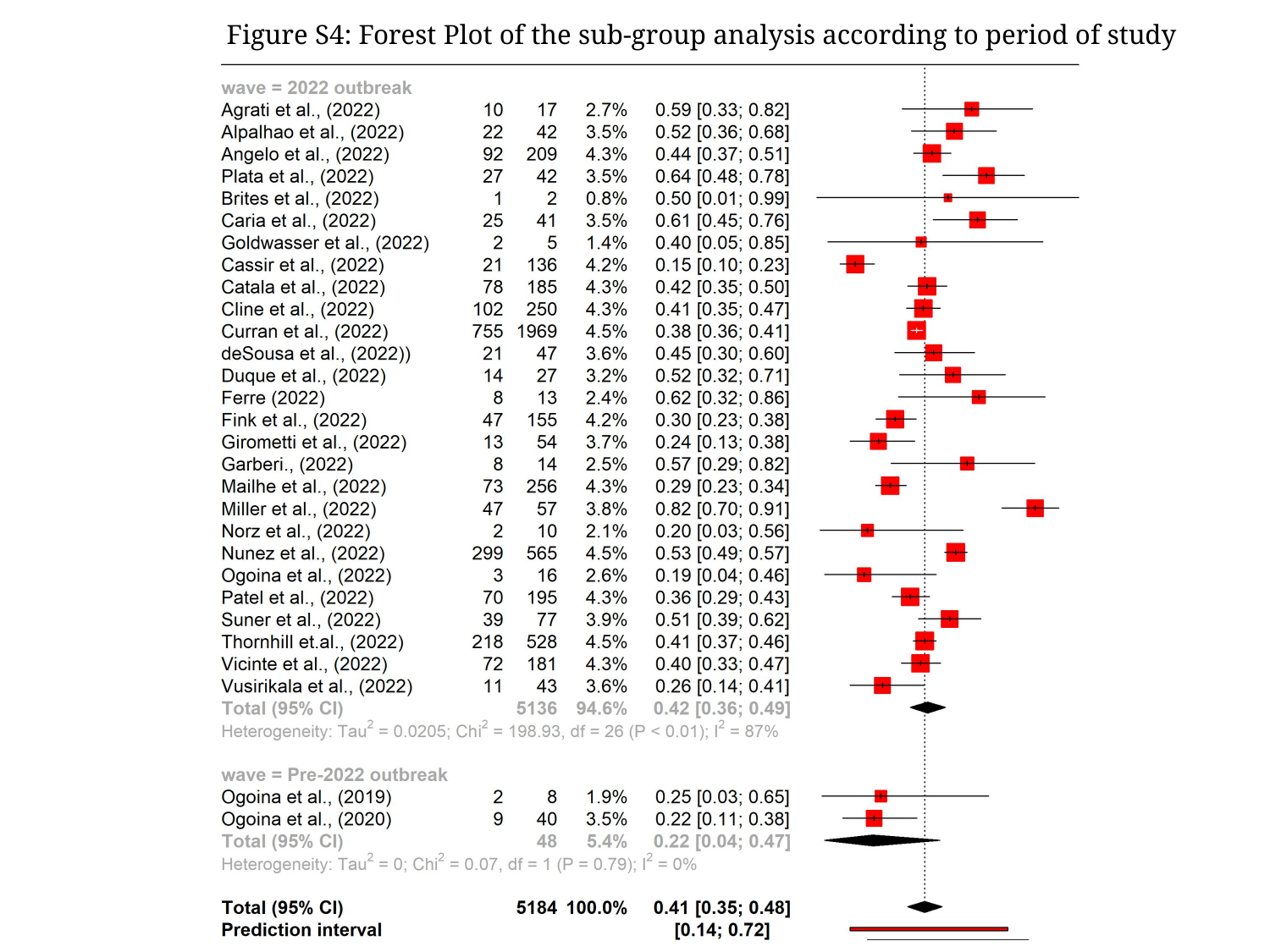

Figure S4: Forest Plot of the sub-group analysis according to period of study

## Slide 5
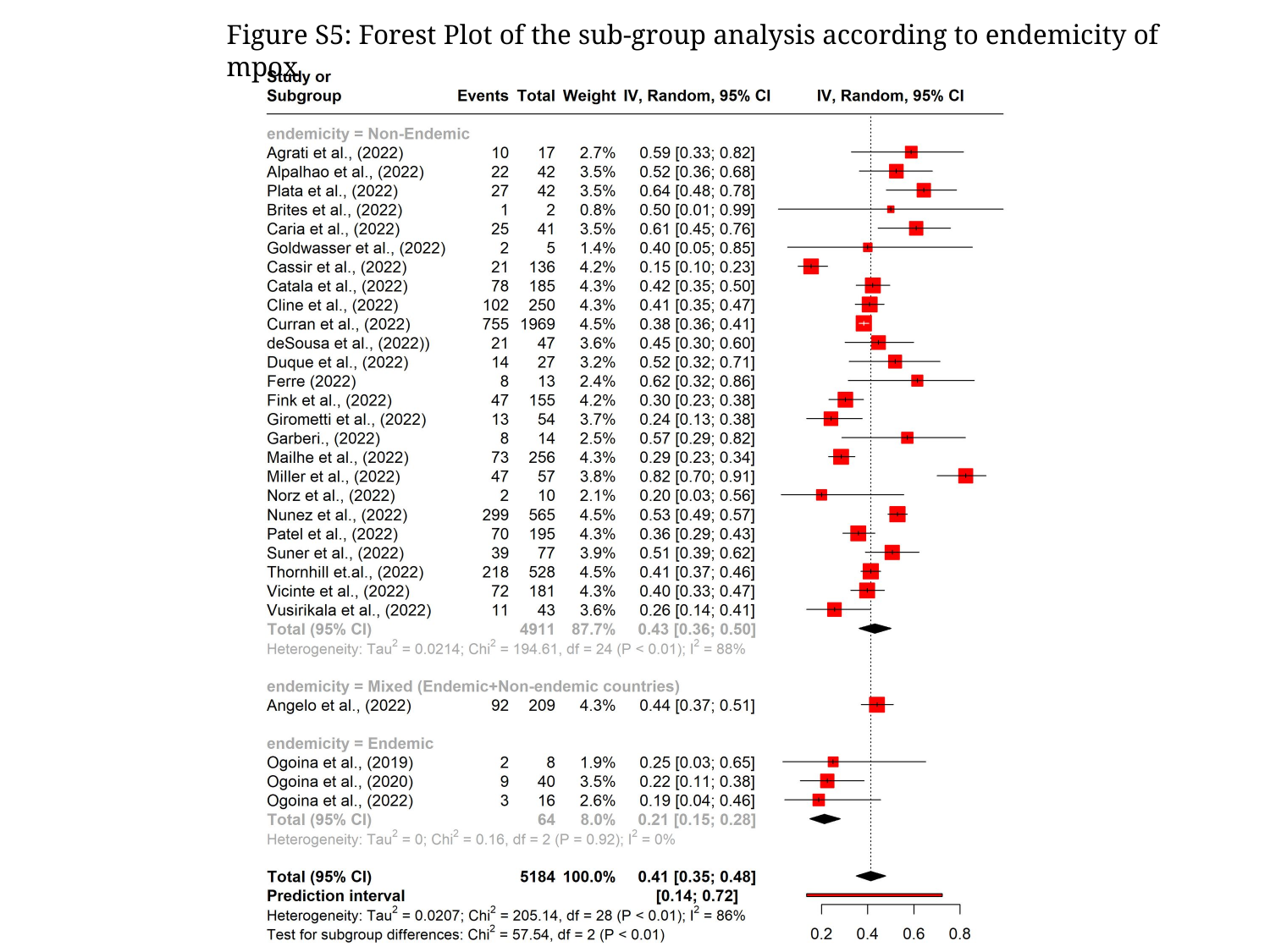

Figure S5: Forest Plot of the sub-group analysis according to endemicity of mpox

## Slide 6
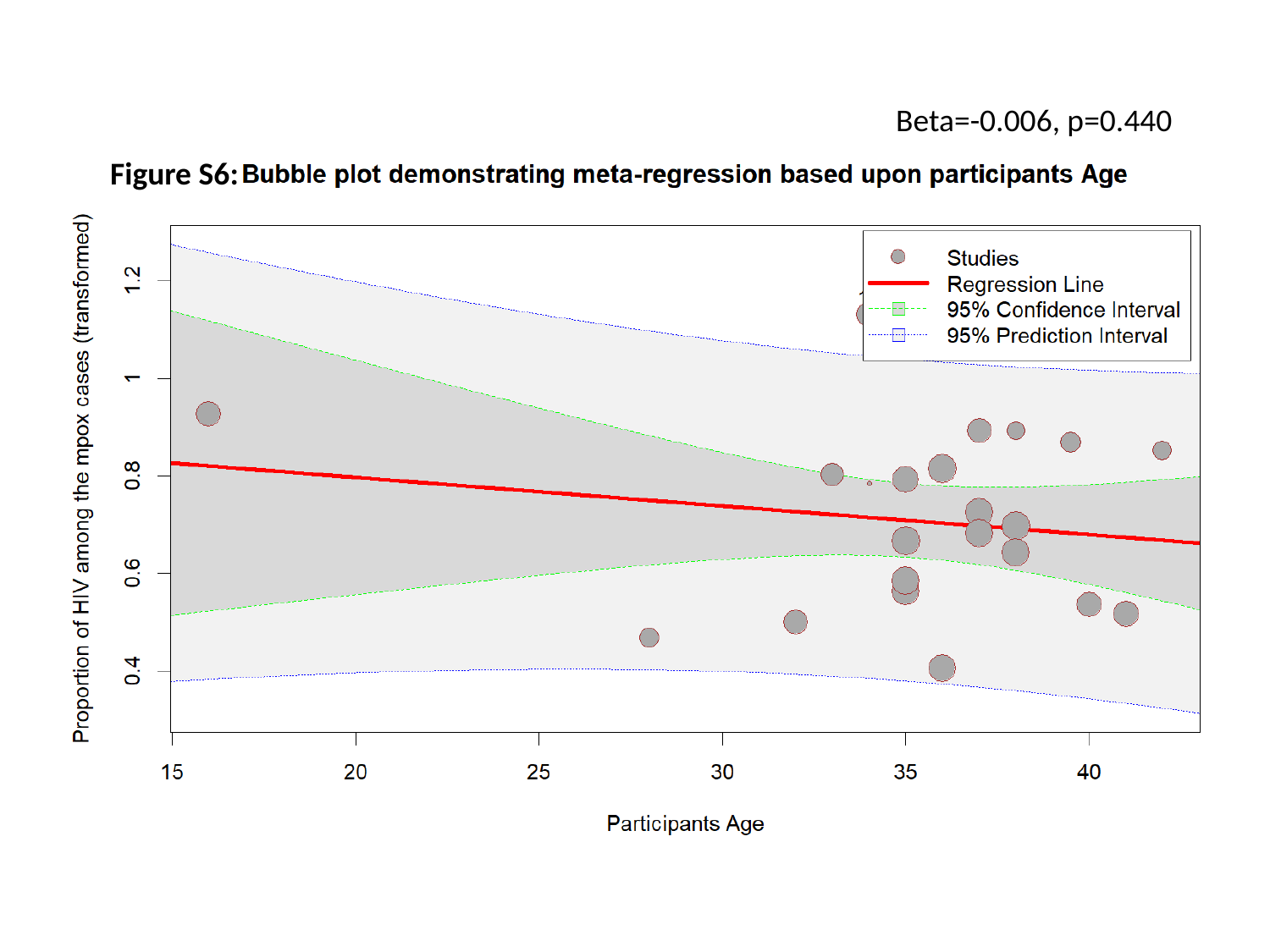

Beta=-0.006, p=0.440
Figure S6:

## Slide 7
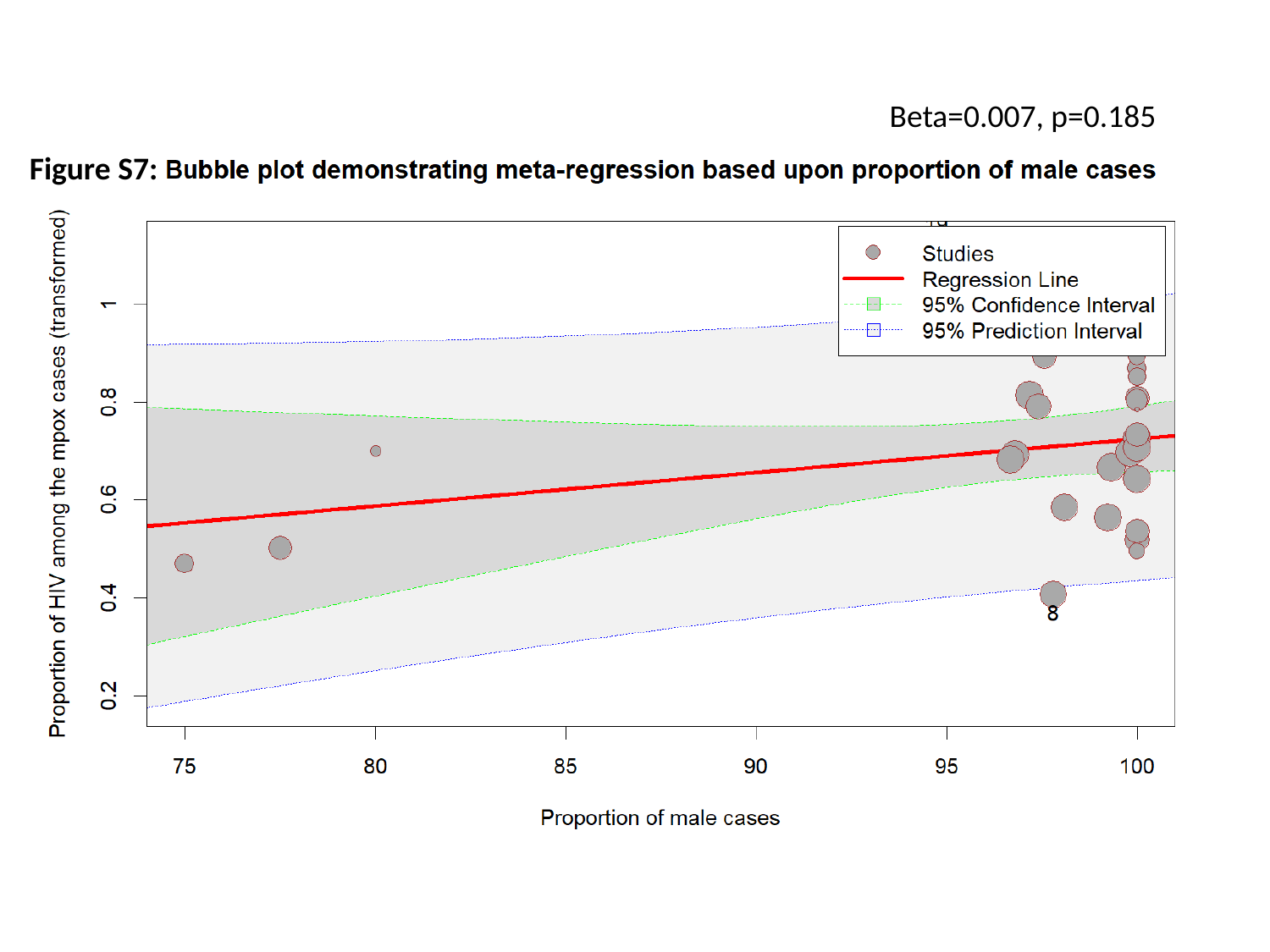

Beta=0.007, p=0.185
Figure S7:

## Slide 8
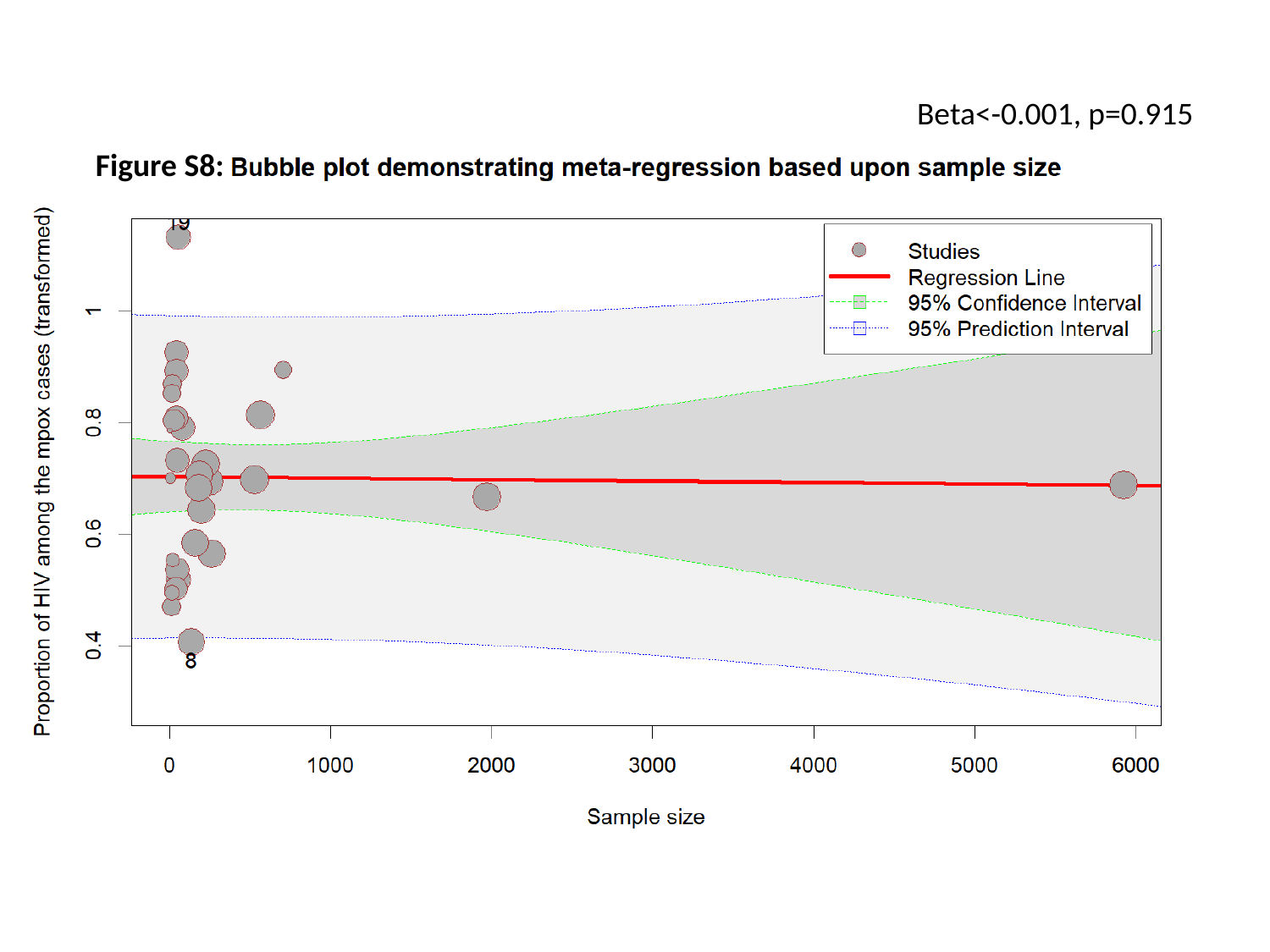

Beta<-0.001, p=0.915
Figure S8:
